# Sirtuins as regulators of mammalian aging

**DOI:** 10.18632/aging.100478

**Published:** 2012-08-15

**Authors:** Shoshana Naiman, Yariv Kanfi, Haim Y. Cohen

**Affiliations:** The Mina & Everard Goodman Faculty of Life Sciences, Bar-Ilan University, Ramat-Gan 52900, Israel

Since the discovery that overexpression of yeast Sir2 deacetylase extends lifespan by as much as 30% over a decade ago [1], much effort has been invested in researching whether this effect is conserved in higher organisms as well. Indeed, in wormsand flies, two separate groups found that SIR2 extended lifespan as well, by 50% and 18%, respectively [1].

In parallel to the work in worms and flies, researchers were trying to make headway in the role of sirtuins in higher organisms. There are seven mammalian homologs to the yeast Sir2, SIRT1-SIRT7. SIRT1 is the most well-researched and has been shown to regulate metabolism and age-related diseases. However, SIRT1 overexpression did not increase lifespan [2], although this was said to be due to the relatively weak expression of the transgene. Therefore, a role for sirtuins in regulating lifespan of mammals looked bleak.

Despite the controversy surrounding sirtuins and longevity, there has never been any doubt that mammalian sirtuins are important regulators of health and disease [3]. Previous results from our lab have shown SIRT6 to be involved in the calorie restriction response [4], and demonstrate that SIRT6 overexpression in mice protects against diet-induced obesity and its metabolic consequences [4]. These results, along with data that SIRT6 knockout mice display a premature aging-like phenotype[5], prompted us to turn towards SIRT6 as a potential regulator of mammalian aging.

Over the course of three years, we measured the lifespan of mice overexpressing exogenous SIRT6 (MOSES). This study was performed in two separate lines from distinct founders, to ensure that the random integration of the transgene into the genome did not influence the results. We found that the gene insertion in both lines did not disrupt any neighboring genes, and results were similar in both lines. In this way we overcame the issue of site-specific integration, which was previously shown to be a problem in sirtuin studies [6]. Additionally, we chose to work with a mixed background, to ensure no strain-specific effects.

Strikingly, both male MOSES lines had significant mean and median lifespan extension, of 14.5% and 9.9% [7]. Even more interesting, there was no lifespan extension in either female lines examined, attesting to a gender-specific role for SIRT6. Additionally, male MOSES displayed improved glucose tolerance in comparison to their wild type littermates. We therefore went on to perform whole-gene microarray and pathway analysis, to elucidate which genes and pathways were altered. Significantly, the IGF-1 signaling pathway was decreased in males only. A reduction in IGF-1 is an integral factor in increasing lifespan in worms, flies, and mice [8]. We then tested the major metabolic tissues to see whether SIRT6 decreased IGF-1 signaling. Surprisingly, we found significantly reduced IGF-1 signaling in the white adipose tissue (WAT) of male mice. These tissue-specific effects were not seen in the muscle or liver, nor were they seen in female mice. Altogether, we found SIRT6 has an integral role in IGF-1 signaling in the fat tissues of male mice, which could potentially explain the longevity seen in male mice overexpressing SIRT6.

For the first time, we have shown evidence for a sirtuin to extend mammalian lifespan. Notably, these results were male-specific, and caused a partial “feminized” effect on the IGF-1 signaling in males. Indeed, very few genes were differentially regulated in females, while over 80 were significantly changed in males. Interestingly, calorie restriction and starvation have previously been shown to have an overall feminized effect, and 30% of differentially expressed genes in these treatments also changed in male SIRT6 transgenic mice [9]. While the exact cause of this feminization shift is unknown, these results may well lead us to the deeper mystery of why females live longer than males in this background.

Additionally, of interest is the fact that alterations in the IGF-1 pathway were found in the WAT tissue. This tissue has garnered attention in recent years, as mice with insulin receptor knockout in adipose tissue displayed increased lifespan [10]. We are currently in the process of researching the exact mechanism of this tissue-specific regulation by creating tissue-specific SIRT6 overexpression.

These exciting results in the sirtuin field have great potential to lead to new medical discoveries, to combat the increasing occurrence of the metabolic syndrome and other age-related diseases. In other words, with SIRT6 around to counteract the effects of high-fat diet and aging, we can have our cake, and eat it too.
